# Case of Early Pregnancy

**Published:** 1896-04

**Authors:** T. J. Mitchell

**Affiliations:** Locust Grove, Ga.


### CASE OF EARLY PREGNANCY.

LOCUST GROVE, GA., February 3, 1896.

*Atlanta Medical and Surgical Journal:*

I have had filed in my office for more than twenty years the following case which I now wish to report: Fanny Allen, colored, born July 17, 1860. Was first confined (girl) January 11, 1872. Again confined (twin boys) July 18, 1873. The woman, or rather girl, thus became a mother at the early age of eleven years, five months, and twenty-three days, and gave birth to twins at the age of thirteen years, one month, and fifteen days.

I would further state the children were of good size, lived and grew off well, and that the mother's pains or suffering in labor appeared to be less than common. The mother was born and raised in Spalding county. T. J. MITCHELL, M.D.

---

A RECENT legal decision declares that a consulting physician called in by the attending physician, for the purpose of consultation as to the treatment of the case, may recover his fees from the patient.—*Food.*

---

"I HAVE more than I can attend to," said the young doctor to the old practitioner. "I had to get up five times last night." "Why don't you get some insect powder?" was the only reply.

